# Insights into the regulatory function of the *ɛ* subunit from bacterial F-type ATP synthases: a comparison of structural, biochemical and biophysical data

**DOI:** 10.1098/rsob.170275

**Published:** 2018-05-16

**Authors:** Alexander Krah, Mariel Zarco-Zavala, Duncan G. G. McMillan

**Affiliations:** 1 School of Computational Sciences, Korea Institute for Advanced Study, 85 Hoegiro Dongdaemun-gu, Seoul 02455, Republic of Korea; 2 Department of Applied Chemistry, Graduate School of Engineering, The University of Tokyo, Tokyo 113-8656, Japan; 3 Department of Biotechnology, Delft University of Technology, van der Maasweg 9, Delft 2629 HZ, The Netherlands

**Keywords:** F-type ATP synthases, *ɛ* subunit, regulation, hydrolysis of ATP, bacterial

## Abstract

ATP synthases catalyse the formation of ATP, the most common chemical energy storage unit found in living cells. These enzymes are driven by an electrochemical ion gradient, which allows the catalytic evolution of ATP by a binding change mechanism. Most ATP synthases are capable of catalysing ATP hydrolysis to varying degrees, and to prevent wasteful ATP hydrolysis, bacteria and mitochondria have regulatory mechanisms such as ADP inhibition. Additionally, *ɛ* subunit inhibition has also been described in three bacterial systems, *Escherichia coli*, *Bacillus* PS3 and *Caldalkalibacillus thermarum* TA2.A1. Previous studies suggest that the *ɛ* subunit is capable of undergoing an ATP-dependent conformational change from the ATP hydrolytic inhibitory ‘extended’ conformation to the ATP-induced non-inhibitory ‘hairpin’ conformation. A recently published crystal structure of the F_1_ domain of the *C. thermarum* TA2.A1 F_1_F_o_ ATP synthase revealed a mutant *ɛ* subunit lacking the ability to bind ATP in a hairpin conformation. This is a surprising observation considering it is an organism that performs no ATP hydrolysis *in vivo*, and appears to challenge the current dogma on the regulatory role of the *ɛ* subunit. This has prompted a re-examination of present knowledge of the *ɛ* subunits role in different organisms. Here, we compare published biochemical, biophysical and structural data involving *ɛ* subunit-mediated ATP hydrolysis regulation in a variety of organisms, concluding that the *ɛ* subunit from the bacterial F-type ATP synthases is indeed capable of regulating ATP hydrolysis activity in a wide variety of bacteria, making it a potentially valuable drug target, but its exact role is still under debate.

## 1. Introduction

All organisms require ATP, a universal chemical energy storage unit, to carry out and maintain cellular functions. ATP synthases are found in almost all kingdoms of life and are the main ATP synthesizing enzymatic machineries in aerobically growing bacterial, archaeal and eukaryotic cells. Recently, the F-type ATP synthase has been shown to be a novel attractive drug-target against *Mycobacterium tuberculosis* [1,2], a causative agent of tuberculosis. Owing to different mechanistic modes of regulation between bacterial and eukaryotic respiratory systems [3], the F-type ATP synthase may be an attractive target for novel antimicrobial compounds.

Bacterial ATP synthases comprise the soluble F_1_ domain [4], harbouring a *α*_3_*β*_3_ hexameric assembly, the *γ* subunit central stalk, the *ɛ* subunit and the *δ* subunit. The *γ* and *ɛ* subunits form a central drive-shaft, connecting the soluble F_1_ domain to the membrane-embedded F_o_ domain [5]. Notably, the *ɛ* subunit of bacteria is the *δ* subunit in mitochondria, but with a divergent function [5]. The membrane-bound F_o_ domain harbours a *c*-ring, where the number of *c* subunits varies from 8 to 15 among different organisms, yet remains invariant within individual organisms [6–10], the *a* subunit, which is horizontally aligned to the c-ring [11,12], and the dimeric *b*_2_ subunit [13]. The *b*_2_ dimer forms a peripheral stalk, connecting the F_o_ domain with the *α*_3_*β*_3_ catalytic hexamer via the *δ* subunit in bacteria [14–16], or the oligomycin-sensitive conferral protein (OSCP) subunit in mitochondrial ATP synthases [11,12,17,18]. A proton- or sodium-motive force drives the rotation of the membrane-embedded *c*-ring [19] versus the membrane-embedded stator subunits *a* and *b*_2_ [11,12]. The transported ion is dependent on the selectivity of the binding site structure in the *c*-ring [20]. In the ATP synthesis direction of *c*-ring rotation (clockwise), a single revolution of the *c*-ring causes a single revolution of the *γ* subunit, which in turn induces conformational changes in all three *αβ* subunits. The net result of this rotation is the catalysis of three ADP and three inorganic phosphate molecules (P_i_) into three ATP molecules [21] coupled to the translocation of either H^+^ or Na^+^ across the cytoplasmic membrane [22]. In addition, this enzymatic reaction can be driven in the reverse direction (anti-clockwise), hydrolysing ATP [23] and pumping ions into either the periplasm or the P-side of the membrane.

The regulation and prevention of ATP hydrolysis is of critical importance, and bacteria and mitochondria have adapted diverse mechanisms of regulation. To date they have been shown to share a common, yet poorly described Mg-ADP hydrolysis inhibition form of regulation [24,25]; however, they have also developed other more distinct, organism-specific mechanisms. In mitochondria, ATP hydrolysis is controlled by the intrinsic regulatory protein IF_1_ [26], while most bacteria have been proposed to be regulated via a conformational transition of the *ɛ* subunit [27]. The exceptions to this are α-proteobacteria, which are regulated by the *ζ* subunit [28–30]. A recent review comparing the different regulatory mechanisms can be found elsewhere [3]. Owing to the unique function of the *ɛ* subunit in bacteria that is not present in higher eukaryotes and its potential as a drug target, the core focus of this article is the role of the *ɛ* subunit in bacterial F_1_F_o_ ATP synthase ATP hydrolysis regulation.

The *ɛ* subunit harbours two domains. The N-terminal domain (NTD) is a rigid β-sheet domain, while the C-terminal domain (CTD) comprises two α-helices connected by a flexible linker [31]. The CTD has been frequently described as having a dynamic nature, with the ability to change conformation depending on the presence or the absence of various concentrations of ATP [32–35]. When these two helices are parallel (i.e. in a ‘down-state’), both spatially localized to the rigid β-sheet domain, the *ɛ* subunit is described to be in the contracted ‘hairpin conformation’, a state which is able to be induced by ATP binding [31,36–39]. Conversely, when these two helices are in series (i.e. in a ‘rod’), spatially distant from the rigid β-sheet domain, yet parallel to the *γ* subunit, and reaching into the *α*_3_*β*_3_ catalytic hexamer (i.e. in an ‘up-state’), the *ɛ* subunit is described to be in the ‘extended conformation’ [40–42]. In the light of the potential interactions the *ɛ* subunit may have in each conformation, and the kinetic effects of various mutants, the extended and hairpin conformations are frequently referred to as the ‘inhibitory’ and ‘non-inhibitory’ states, respectively, regarding the ATP hydrolysis ability of the enzyme. In the authors' view, until we fully understand the mechanism, it is prudent to simply refer to these conformations by their shape (‘hairpin’ and ‘extended’) until a solid consensus can be reached on the functional mechanism, or whether the strength of the influence of the regulatory role is simply more species-dependent than previously assumed.

Structures of isolated *ɛ* subunits from *E. coli* [36,38], *Bacillus* PS3 [39] and *Thermosynechococcus elongates* BP-1 [31] show that the *ɛ* subunit adopts a hairpin conformation. Conversely, an NMR structure of the c-terminal helix (CTH) truncated *ɛ* subunit (*ɛ*Δ^103–120^) from *Mycobacterium tuberculosis* was found in the extended conformation in the absence of ATP [43], implicating the CTH in a critical role for maintaining the hairpin conformation. This suggests that the *ɛ* subunit is capable of a dynamic conformational movement. Yet more clearly indicative of conformational dynamics was an *E. coli* F_1_ (EF_1_) *ɛγ* subunit structure, where the *ɛ* subunit was found in a ‘half-extended’ conformation [44]. In structures of the whole F_1_ domain, the *ɛ* subunit has been found in either the extended or hairpin conformation, as shown in the F_1_ domain from *Bacillus* PS3 [41], *Paracoccus denitrificans* [15], the F_1_ [40] and F_1_F_o_ [16] from *E. coli*, and the F_1_ from *Caldalkalibacillus thermarum* TA2.A1 [45]. However, contrary to the body of evidence supporting an ATP-mediated hairpin conformation, a *C. thermarum* TA2.A1 *ɛ* subunit lacking the ability to bind ATP was also found in the hairpin conformation [45]. Taken together, structural studies have revealed a number of ‘snapshots’ from various species, but no complete picture currently exists.

ATP hydrolysis is prevented selectively in several bacterial F_1_F_o_ ATP synthases studied to date. In three *Bacillus* species [46–48], *P. denitrificans* [49] and two mycobacterial species (*M. smegmatis* and *M. bovis*), ATP hydrolysis is selectively prevented [50–52]. It has long been proposed that the extent of ATP hydrolytic inhibition is due to the binding affinity of the *ɛ* subunit for ATP, a proposal supported by the observation that the *ɛ* subunit from different organisms binds ATP with different affinities. Isolated *ɛ* subunits have widely ranging binding affinities, from *Bacillus* PS3 with an apparent binding constant of 4 µM [53,54] to 2 mM for *Bacillus subtilis* [55] and 22 mM for *E. coli* [39]. ATP binding assays have also been carried out for whole F_1_ complex (*α*_3_*β*_3_*γɛ*) from *T. elongatus* BP-1 [56] and various mycobacterial species [43], but ATP binding was not observed in the measured concentration range.

In the light of these data, the regulatory role of the *ɛ* subunit in bacterial systems remains controversial. In this study, we compare available biochemical, biophysical and structural data from different bacterial organisms to help clarify the state of the field.

## 2. Current evidence on the regulatory role of the *ɛ* subunit

### 2.1. Biophysical and biochemical experiments indicate a regulatory mechanism dependent on the *ɛ* subunit

#### 2.1.1. Escherichia coli

To the best of our knowledge, the first suggestion that the *ɛ* subunit had an inhibition role of ATP hydrolysis in an *in vivo* setting was described for the EF_1_ domain [42]. Biochemical cross-linking experiments showed that the *ɛ* subunit from *E. coli* could be covalently attached to the *α*_3_*β*_3_ assembly [57,58], indicating an extended conformation, and was later resolved in an F_1_ crystal structure [40]. Interestingly, in a different study, the same authors observed that the ɛ-β crosslink (*ɛ*S108C-βD380C) occurs less frequently in the presence of ATP compared with the presence of ADP or the competitive inhibitor AMP-PNP [59]. This led to the hypothesis that the decreased crosslinking might be caused by decreased binding affinities of ADP and AMP-PNP to the CTD of the *ɛ* subunit, thus allowing the *ɛ* subunit to stay in the extended conformation.

The conformational transition of the *ɛ* subunit has also been observed using Förster resonance energy transfer (FRET) experiments [60]. Although this was not immediately evident in initial experiments of the same group [61], this movement has because been independently confirmed using single-molecule FRET studies, supporting the idea of a conformational transition [62]. In single-molecule rotation experiments, the *ɛ* subunit increases the duration of the pause during the rotational motion of the ATP synthase in hydrolysis direction [63]. *ɛ*S108 (S108A/D) and *ɛ*Y114 (Y114A) mutations interact with *β*E381:O*ɛ*x and *γ*G85:O, respectively (shown in the crystal structure [40]), reducing the inhibitory effect on ATP hydrolysis by the *ɛ* subunit [64]. Cross-linking was also observed between *β*E381C/*β*S383C and *ɛ*S108C, also suppressing ATP hydrolysis activity [65]. This study is supported by further studies describing cross-linking between *ɛ*A117C and *c*Q42C (revealing the *ɛ* subunit in a hairpin conformation) and between *ɛ*A118C and *γ*L99C (revealing the *ɛ* subunit in an extended conformation) [58]. To the best of our knowledge, this is the first report confirming that the *ɛ* subunit could exist in two distinct structural states. Furthermore, it has been claimed that inhibition caused by the *ɛ* subunit is separate from Mg-ADP inhibition [66], and a dynamic transition between the released and tightly bound auto-inhibited state by the *ɛ* subunit has been proposed [66,67]. It has also been proposed that the *ɛ* subunit fine-tunes fundamental steps in the ATP synthesis direction [34,68].

Lastly, it should be mentioned that the ATP binding affinity of the isolated *ɛ* subunit from EF_1_ (22 mM) [39] is below the average physiological bulk ATP concentration in *E. coli* cells (1.54 mM) [69], making it seem less likely that ATP has a regulatory function by binding to the *ɛ* subunit under physiological conditions. Nonetheless, we cannot discount the potential influence of localized ATP pools before diffusion away from the ATP synthase, a difficult phenomenon to study with any degree of accuracy.

#### 2.1.2. *Bacillus* PS3

Both the isolated *ɛ* subunit from *Bacillus* PS3 [39,70] and the *ɛ*γ-subunit complex [71] have been shown to bind ATP. Mutations in the CTH of the *ɛ* subunit from *Bacillus* PS3 [39] result in decreased ATP binding affinity, supporting the notion that the *ɛ* subunit from *Bacillus* PS3 binds ATP [53]. Additionally, using the F_1_ complex (TF_1_), Iino *et al.* [32] monitored extended/hairpin conformation transitions using FRET between labelled residues in the *ɛ* and/or *β* subunits. Importantly, this study also revealed that the *ɛ* subunit has sub-millimolar affinity to ATP at close to the optimal growth temperature of *Bacillus* PS3, suggesting that the *ɛ* subunit is an ATP concentration sensor *in vivo* [32]. However, the time taken for these conformational shifts suggests that the *ɛ* subunit exerts a slow switch-like regulation of ATP hydrolysis, rather than a rapid movement. Furthermore, Iino *et al*. [32] observed that the dependence of the ATP synthetic activity of *E. coli* F_1_F_o_ wild-type (WT) versus Δ*ɛ* C-terminus on ΔpH and Δ*Ψ* was similar, suggesting that the loss of the *ɛ* subunit CTH is not rate-limiting for ATP synthesis [32]. Supporting this, single-molecule rotation measurements of TF_1_ revealed that in the presence of low ATP concentration (200 nM), comparatively more numerous and extended pauses in rotation were observed in the presence of the WT *ɛ* subunit, than in the absence. However, at a higher ATP concentration (2 µM) the *ɛ* subunit presence/absence had negligible effect on enzyme kinetics. This study provides support for the slow conformational shift regulation model, and provides insight that the extended conformation may inhibit ATP hydrolysis in TF_1_. However, in a TF_1_ harbouring an *ɛ* subunit truncation mutant, *ɛ*Δ^CTD^, (a stop codon after *ɛ*D87), no obvious differences were observed in rotation from the WT TF_1_ complex [72].

Interestingly, the crystal structures of the isolated *ɛ* subunit [39] and in the TF_1_ complex [41] have both been shown to be capable of taking both extended and hairpin conformations. In the case of the isolated *ɛ* subunit, in the presence of ATP, the overall structure is very similar to the isolated *E. coli ɛ* subunit (a hairpin conformation). In the absence of ATP, the NMR structural data were relatively poorly resolved; however, on the basis of the dihedral ϕ/*ψ* angles, the authors were able to discern that a proportion of the molecules formed a long single helix, supporting the notion that the hairpin structure can transition into an extended helical structure, and that that extension of the structure is what drives the inhibition of ATPase hydrolysis at low ATP concentrations [39]. What this study also revealed is that the conformation shift is likely to be dynamic, supporting a previous finding by Iino *et al.* [32] and the later observations of Tsumuraya *et al.* [72]. Recently, the TF_1_ crystal structure (*α*_3_*β*_3_*γɛ*) was solved, where the *ɛ* subunit was found in the extended conformation, structurally confirming the extended helical structure in a physiological structural context [41]. The crystal contacts, like the *ɛ* subunit extended conformation *E. coli* structure, strongly support the notion that the extended conformation inhibits rotation in the hydrolysis direction. Furthermore, mutagenesis experiments revealed that mutations in either the DELSDED motif of the *β* subunit (DELSEED in mammalian F_1_), or the terminal helix of the CTD from the *ɛ* subunit, resulted in an increase in the ability of TF_1_ to catalyse ATP hydrolysis, supporting the proposed role of the *ɛ* subunit as an inhibitor of ATP hydrolytic activity. This study also suggests that ATP hydrolysis inhibition caused by the *ɛ* subunit may be due to an electrostatic interaction between the CTH of the *ɛ* subunit and the DELSDED motif of the TF_1_ *β* subunit [73]. In addition, there is a growing body of evidence supporting the notion that these conformational changes may also be dependent on proton motif force (pmf) [33,74].

It has been proposed that the directionality of the *γ* subunit directs the conformational state of the *ɛ* subunit. For this to be feasible, the *c*-ring must transmit a torque larger than the thermodynamic equilibrium to the *γ* subunit by an increased pmf (approx. 400 mV) to revoke the *ɛ* subunit extended conformation and initialize ATP synthesis by the F_1_ complex [75]. In agreement with this theory, the mutation of critical ATP binding residues, E83 and R92 [53], did not influence the transition from the extended to hairpin conformation. This finding suggests that the nucleotide occupation in the catalytic binding site in the *β* subunit induces the conformational change of the *ɛ* subunit [32] to a half-extended conformation, while in a last step ATP may bind to the *ɛ* subunit, trapping the *ɛ* subunit in a contracted ATP-bound state. This contracted ATP-bound structure stabilizes the hairpin conformation [35,76]. The justification that has been proposed is that during ATP hydrolysis, the contracted ATP bound state is necessary to prevent uncoupling between ATPase activity and H^+^ pumping [74]. Furthermore, the *ɛ* subunit has been proposed to decrease ADP binding affinity to the catalytic site (*α*_3_*β*_3_) [77], taking a different mode of action from Mg-ADP inhibition of ATP hydrolysis [78], relieving the inhibition state [79]. However, this is difficult to unravel as the extended pauses observed during rotation experiments were at the same angular positions as Mg-ADP inhibition [72].

#### 2.1.3. *Caldalkalibacillus thermarum* TA2.A1

The thermoalkaliphilic bacterium *C. thermarum* TA2.A1 grows at 65°C and at pH levels between 7.5 and 10.2 on fermentative substrates [80,81], with highest growth rates aerobically at alkaline pH (9.5) [82]. The pH-dependent growth is caused in part by inefficient ATP synthesis at pH values below 8.5 caused by a lysine residue (K180) in the *a* subunit of the ATP synthase, allowing proton translocation and thus ATP synthesis optimally under alkaline pH conditions [83].

Given the energetically hostile conditions required for *C. thermarum* TA2.A1 growth, it is essential that such an organism conserve ATP. Therefore, it is unsurprising that the ATP hydrolysis activity of the F_1_F_o_ or F_1_ ATP synthase from *C. thermarum* TA2.A1 is suppressed under physiological conditions, identified by biochemical [48,82,83] and single-molecule experiments [84]. Keis *et al.* [85] demonstrated that in the presence of a low ATP concentration (50 µM), the WT F_1_F_o_ and F_1_ have negligible ATPase activity, while at high ATP concentrations (2 mM) ATP hydrolysis was observed. This hints at a conformational change of the *ɛ* subunit from the extended to the hairpin conformation upon ATP binding to the catalytic *β* subunit (as observed in *Bacillus* PS3 [32]) or ATP binding to the *ɛ* subunit itself. Conversely, in a recent study, regardless of ATP concentration, negligible ATP hydrolysis was observed in the absence of chemical or mechanical activation [84].

ATP hydrolysis activity is not inhibited if ATP binding residues in the CTH (residues R123 and R127), or if proposed non-ligand binding residues R116, H117, K118 and R119 (TA2F_1_ Δ*ɛ*^6A^), are mutated to alanine [85], while mutating R116, H117, K118 and R119 simultaneously to alanine still prevented ATP hydrolysis activity at low ATP concentration (50 µM) [81]. Collectively, these data suggest that residues *ɛ*R123 and/or *ɛ*R127 bind to the *α*_3_*β*_3_*γ* complex, and could potentially serve to stabilize an inhibitory extended conformation, akin to the interaction between *ɛ*K123 and *β*D372, or the salt bridge between *ɛ*R122 and *β*D382 in the *ɛ* subunit from *E. coli* and *Bacillus* PS3, respectively. In contrast to these clear observations, a mixed effect was observed upon trypsin treatment, where the WT TA2F_1_F_o_ and TA2F_1_ complexes are protected by trypsin digestion compared with the TA2F_1_ Δ*ɛ*^6A^ mutant, in which the *ɛ* subunit was completely degraded. We would like to reinforce the point that in spite of *the very same mutant being more active in ATP hydrolysis* than the WT TA2F_1_, no further activation of ATPase activity was observed [85]. The reason for this remains unclear.

The recent structures of the WT TA2F_1_ and a mutant, in which the ATP binding site is lost (D89A/R92A), revealed the *ɛ* subunit in a similar hairpin conformation [45], akin to that of the *E. coli* [36,38] and *P. denitrificans* [15] *ɛ* subunits. Notably, the *E. coli* and *Bacillus PS3 ɛ* subunits have also been solved in the extended conformation [40,41], so while it is curious that the TA2F_1_ D89A/R92A mutant resulted in a hairpin conformation, we know from the more vigorously studied EF_1_ that this is not entirely unexpected as the conformations are clearly dynamic in nature [44]. Having noted this, theoretically, the TA2F_1_ D89A/R92A mutant should lack the ability to bind ATP, and if this is the regulator inducing the formation of a hairpin conformation, one would expect to find an extended conformation *ɛ* subunit structure. However, this study clearly demonstrates that the lack of ATP presence, or, more directly, the lack of an apparent ATP-binding motif, appears not to necessarily mean the *ɛ* subunit will definitively adopt the extended conformation at all times, if indeed it does in TA2F_1_.

In the context of the previously discussed organisms, the structural role of *ɛ*R126 (*Bacillus* PS3) and *ɛ*Q127 (*E. coli*, numbering as in the deposited crystal structure) are not clear as these residues are not resolved in the extended conformation in *Bacillus* PS3 *ɛ* subunit [41], nor are there any obvious interactions with the *α*_3_*β*_3_*γ* interface in the *E. coli ɛ* subunit [40]. However, the role of these residues in the *ɛ* subunit from *C. thermarum* TA2.A1 in the extended conformation cannot be assumed, as the *ɛ* subunit was neither resolved [86] nor found to reside in the extended conformation [45] in the conditions used in the presently available crystal structures.

Lastly, while the focus of this review is on *ɛ* subunit regulation, it should be mentioned that the *γ* subunit has a potential role in ATP hydrolysis regulation in TA2F_1_. A mutation changing ^8^KRRIR^12^ residues in the *γ* subunit of TA2F_1_ to ^8^QQQIQ^12^ residues (TA2F_1_*γ*^Q4^) resulted in a similarly partially active hydrolytic enzyme with similar ATP hydrolysis kinetics to the TA2F_1_ Δ*ɛ*^6A^ mutant previously mentioned [86].

### 2.2. Structural properties of the *ɛ* subunit from bacteria

#### 2.2.1. Comparison of structural features of isolated *ɛ* subunits in the hairpin conformation

Although certain crystallographic features of the *ɛ* subunit have been previously discussed in the context of the biochemical and biophysical section of this manuscript, such is the complexity of the topic that a dedicated section must be presented to give the full picture on this topic.

First, we compare the isolated *ɛ* subunits from different organisms in the context of their highly varied ATP binding affinities. The crystal structure of the *ɛ* subunit from *Bacillus* PS3 shows a well-defined ATP binding motif comprising interactions of E83, D89 (backbone), R92, R122 and R126 with ATP [39]. However, the crystal structure was solved as a dimer, and thus does not reflect the monomeric presence of the *ɛ* subunit in bacterial ATP synthases. Molecular dynamics simulations have since served to refine models of the ATP binding site, and predict where Mg^2+^ ions bind between ATP:O*α*/O*β* [83], which has also been observed in the structure from *C. thermarum* TA2.A1 [45].

The well-defined binding motif in the *ɛ* subunit from *Bacillus* PS3 enables the protein to bind ATP with an affinity of 4 µM at 25°C [53]. Interestingly, under the same conditions, a R103A/R115A double mutant was capable of binding ATP with two orders of magnitude increased affinity (52 nM) [54]. This was proposed to be caused by an increased number of hydrogen-bonds between the protein and the ligand due to a structural rearrangement of the ligand binding site [87]. ATP binding affinities of the *ɛ* subunit from *B. subtilis* (2 mM at 25°C) [55], *M. tuberculosis* (ATP binding was not observed in the measured range) [43] and *E. coli* (22 mM at 25°C) [39] have also been reported.

**Figure 1.**
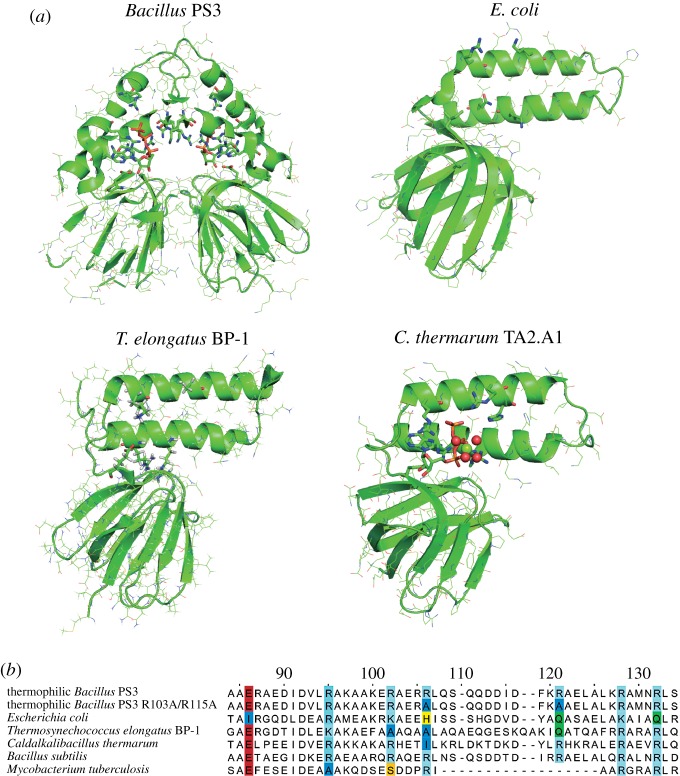
(*a*) Crystal/NMR structures of the *ɛ* subunits from *Bacillus* PS3 (PDB-ID: 2E5Y), *Escherichia coli* (PDB-ID:1AQT), *Thermosynechococcus elongates* BP-1 (PDB-ID: 2RQ6) and *Caldalkalibacillus thermarum* TA2.A1 (PDB-ID: 5HKK—only the *ɛ* subunit is shown; all other F_1_ subunits are omitted) in the hairpin conformation. Known and potential binding residues are highlighted. (*b*) Sequence alignment of the binding site of several *ɛ* subunits from different organisms. The binding site residues are coloured/highlighted. The sequence alignment was created using Jalview [88].

Current structural data (figure 1*a*) and a sequence alignment (figure 1*b*) indicate that the proposed ATP binding site composition controls the ligand binding affinity. In the *ɛ* subunit from *Bacillus* PS3, positively charged residues can be found [39], some of them exchanged by polar and/or hydrophobic amino acids in the α-helical CTD of the *ɛ* subunit from *E. coli* [36,38] or *T. elongates* BP-1 [31] (see also the sequence alignment in figure 1*b*). To derive the reasons for the different binding affinities of ATP to the *ɛ* subunit from different organisms, we have aligned and compared the sequences of *ɛ* subunits from different organisms with known K_d_ to bind ATP (figure 1*b*), and compared them with the most well-described enzyme for these studies, *Bacillus* PS3. When comparing the *ɛ* subunit sequences of *Bacillus* PS3 and *B. subtilis,* there are no differences in the proposed ATP binding motif, yet they differ 500-fold in their ATP binding affinity (4 µM versus 2 mM for *Bacillus* PS3 [53] and *B. subtilis* [55], respectively). This suggests that there are other factors to consider.

It has been previously proposed that an allosteric Mg^2+^ binding site causes a reduction of the ATP binding affinity [89], which is in agreement with the experimentally measured decreasing K_d_ of the *ɛ* subunit R84A mutant from *Bacillus* PS3 [53]. Furthermore, the alignment of the different proposed ATP binding motif residues show that the *ɛ* subunit from *E. coli* harbours four divergences from the *Bacillus* PS3 primary sequence: E83I, R99 K, R122 K and R126Q (in alignment positions 86, 102, 128 and 132, respectively). Mutations of three of these residues (E83, R122 and R126) to alanine have shown a remarkably reduced ability of the *ɛ* subunit to bind ATP from *Bacillus* PS3 [53], while the R99A mutation showed a moderate effect in gel-filtration experiments.

Subtle divergences in proposed ATP binding motifs in protein sequence alignments appear to have significant effects. The *ɛ* subunit from *C. thermarum* TA2.A1 harbours a divergence in the proposed ATP binding motif from the *Bacillus* PS3 sequence in position 103 (R103I in respect to *Bacillus* PS3, position 106 in alignment). Owing to its structural location, this mutation may indeed increase ATP binding affinity, as shown for the R103A/R115A double mutant from the *ɛ* subunit from *Bacillus* PS3 [54]. This proposition remains to be experimentally verified. The *ɛ* subunit sequence from *T. elongates* BP-1 differs from *Bacillus* PS3 at positions 95 and 102, which have been shown to reduce ATP binding affinity [53]; however, as these mutations may not be crucial for ATP binding, the ATP binding affinity might theoretically be expected to be in the higher millimolar range. The *ɛ* subunit from *M. tuberculosis* also harbours mutations in these positions (R92A and R99S; alignment positions 95 and 102, respectively), which were shown to decrease the ATP binding affinity. When arginine residues (R92 and R99) were mutated to alanine residues in the *ɛ* subunit of *Bacillus* PS3, the R92A mutation caused a decreased binding affinity of 40-fold (4 µM of WT versus 160 µM of R92A mutant) [53]. Interestingly, the *ɛ* subunit from *M. tuberculosis* also contains a gap of 16 residues in the CTD, thus missing potentially stabilizing hydrophobic interactions. This gap may cause the lack of any observed hairpin conformation, as indicated by small-angle X-ray scattering (SAXS) observations [43], but definitively highlights this *ɛ* subunit as a unique and promising drug target.

#### 2.2.2. Structural features of the *ɛ* subunit in a state between the hairpin and extended conformations

**Figure 2.**
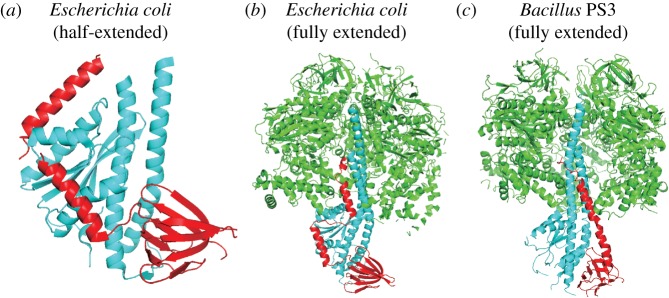
The *ɛ* subunit from *Escherichia coli* in (*a*) the half-extended conformation in the presence of the *γ* subunit (PDB-ID: 1FS0) and (*b*) in the extended conformation in presence of *α*_3_*β*_3_*γ* (PDB-ID: 3OAA), and (*c*) the *ɛ* subunit from *Bacillus* PS3 in the extended conformation in the presence of *α*_3_*β*_3_*γ* (PDB-ID: 4XD7). One *β* and one *α* subunit are omitted for clarity in the extended conformation of the *ɛ* subunit from *E. coli* and *Bacillus* PS3, respectively. Subunits *α*_3_*β*_3_, *γ* and *ɛ* are shown in green, cyan and red, respectively, in all panels.

The first structural evidence showing that the *ɛ* subunit from *E. coli* has dynamic conformational changes was an X-ray structure capturing a half-extended conformation (or half-hairpin) derived from a *γɛ* complex (PDB-ID: 1FS0; figure 2*a*) [44]. This conformation is dramatically different from the other presented structures (isolated *ɛ* subunits), which have been reported to be in the hairpin conformation by NMR (PDB-ID: 1BSN) [37] and X-ray crystallography (PDB-ID: 1AQT; figure 1*a*) [38]. These different conformations of the hairpin conformation (PDB-IDs: 1BSN and 1AQT) and the half-extended state (PDB-ID: 1FS0) indicate that protein–protein interactions are likely to stabilize the extended conformation.

Current ATP fluorescence sensors based on the *ɛ* subunit show that, in the absence of ATP, an extended conformation will be adopted [69,90,91], an observation supported by the finding that the *ɛγ* complex from *Bacillus* PS3 is capable of binding ATP [71]. If the whole F_1_ domain is present during crystallization, a fully extended state of the *ɛ* subunit [40] can be observed in F_1_ from some bacterial species, binding to *α*_3_*β*_3_*γ* and thus preventing rotation in the hydrolysis direction. Interestingly, a recent cryo-EM study revealed that the *ɛ* subunits of all F_1_F_o_ particles from *E. coli* adopt the extended conformation [16]. However, binding conformations of these cryo-EM structures are different from the previously resolved X-ray structures. The structures of the *α*_3_*β*_3_*γɛ* complex from *E. coli* [40] and *Bacillus* PS3 [41] in the extended conformation are shown in figure 2*b*,*c*. Lastly, as the bulk phase ATP concentration in living cells is in the millimolar range (approx. 1.54 mM for the *E. coli* cell cytoplasm [69]), and the binding constant of ATP to the *ɛ* subunit from *E. coli* is 22 mM [39], it would seem logical that the *ɛ* subunit is predominantly in the extended conformation under physiological conditions. However, two factors cannot be excluded: first, that the *ɛ* subunit is in a similar conformation to the half-extended conformation, as the *ɛ* subunit will be released after ATP binding to the *β* subunit [32]; second, the role or possibility of localized ATP pools prior to diffusion into the bulk phase.

#### 2.2.3. Revisiting the crystal structure from *Caldalkalibacillus thermarum* TA2.A1

Recent biophysical [32–35,68], crystallographic [40,41] and cryo-EM [16] data support the conclusion that the two C-terminal helicies of the *ɛ* subunit from different organisms are capable of undergoing a conformational change from a hairpin to an extended conformation. In the crystal structure of the WT TA2F_1_, the *ɛ* subunit is found in the hairpin conformation bound to ATP (PDB-ID: 5HKK), as shown previously for the isolated *ɛ* subunit from *Bacillus* PS3 [39]. However, unexpectedly, the TA2F_1_ D89A/R92A mutant is also found in the hairpin conformation, despite ATP not binding to the *ɛ* subunit in the crystal structure (PDB-ID: 5IK2) [45]. This contradicts our present understanding of the dynamics and regulatory role of the *ɛ* subunit that have been observed from concerted *E. coli* and *Bacillus* PS3 studies. We have made the comparison with *Bacillus* PS3, as this is more closely related, phylogenetically, to *C. thermarum* TA2.A1 than *E. coli*. First, NMR data of TF_1_ showed the structure was difficult to resolve due to dynamic movements, in the absence of ATP [39], perhaps also due to the lack of true resting conformation, but an extended form was indeed revealed. The *C. thermarum* TA2.A1 *ɛ* subunit may simply fall into the hairpin conformation more consistently, providing a tighter trend. This is an interesting, and perhaps unique feature of *C. thermarum* TA2.A1, because we cannot discount the increase in ATP hydrolysis observed with the *γ* subunit mutant TA2F_1_*γ*^Q4^ compared with the native latent WT activity [86]. Second, the structure is consistent with the rotation studies of *Bacillus* PS3, where the WT TF_1_ and TF_1_ *ɛ*^ΔCTH^ also have an identical kinetic profile to the WT enzyme [72].

**Figure 3.**
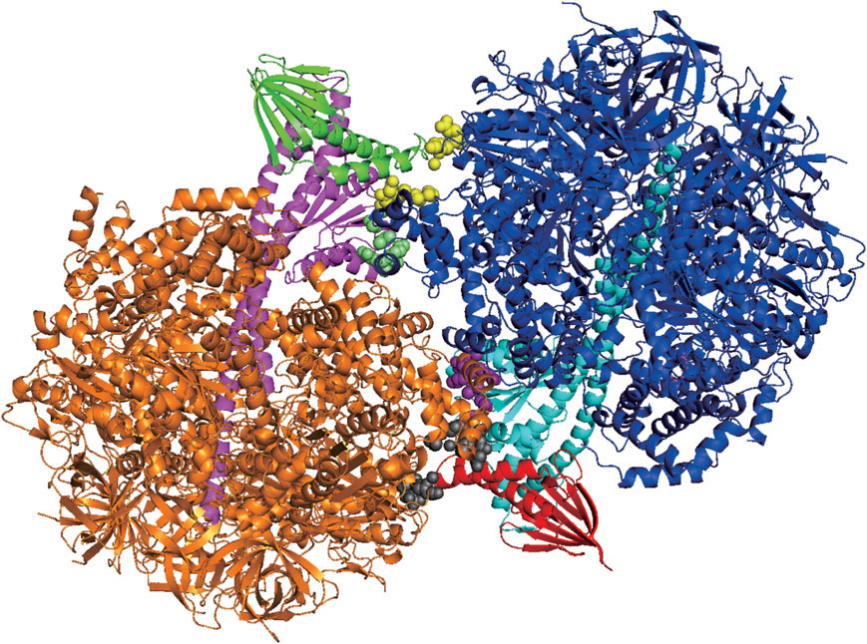
The structural model of TA2F_1_ D92A/R92A mutant, whose *ɛ* subunit apparently does not bind ATP, as deposited in the Protein Database (PDB ID: 5IK2). It can be observed that the *α*_3_*β*_3_ interacts with subunits *γ* and *ɛ* of the other ATP synthase. Subunits *ɛ*, *γ* and *α*_3_*β*_3_ are shown in green (red), magenta (cyan) and orange (blue) in cartoon representation, respectively. Contacts of the *α*_3_*β*_3_ assembly with subunits *ɛ* and *γ* in the distance of 5 Å are shown in yellow (grey) and lime (purple), respectively. This figure was created with PyMOL v. 1.7.0 (www.pymol.org) [92]. A zoom-in for the contacts of monomer A and B is shown in electronic supplementary material, figure S1.

Another possible reason might lie with the technical details in the mutant structure [45]. First, we observe two independently crystallized F_1_ subunits of the ATP synthases in the unit cell, A and B. Second, we observe crucial interactions between the *ɛ* and *γ* subunits of monomer A (spheres; coloured in purple and yellow) with the hexagonal *α*_3_*β*_3_ assembly of monomer B and vice versa (spheres; coloured in grey and lime), as shown in figure 3. Despite the absence of ATP, these interactions may subtly favour the *ɛ* subunit in the hairpin conformation, because the hairpin conformation could also be observed for the ATP-free, monomeric, isolated apo structure of the *ɛ* subunit by NMR spectroscopy [31,36] or X-ray crystallography [38]. Protein–protein interactions, which are prevented in this structure [45] by interactions of the *ɛ* and *γ* of monomer A with *α*_3_*β*_3_ of monomer B, and vice versa, may also be required for the conformational change, as indicated by the crystal structure of the *ɛ*γ-complex from the F-type ATP synthase from *E. coli* [44]. This is a pertinent point when considering the consistent presence of the hairpin conformation in the absence of the *γ* subunit when resolving full-length *ɛ* subunit structures [36,38]. However, to confound the matter, the conformational change from the extended to the hairpin conformation of the *ɛ* subunit in the *γɛ* sub-complex is also induced by binding to ATP [71], and is a dynamic movement, and therefore highly conditional. Clearly, there is still much research to conduct before this can be resolved.

## 3. Conclusion and the 6th antibiotic target space

### 3.1. Regulatory function of the *ɛ* subunit

Considering biochemical and biophysical measurements, and the structural data in the hairpin [36,38,39], extended [16,40,41] and half-extended conformations [44], there are various lines of evidence that support the notion that the *ɛ* subunit of ATP synthases from at least some bacteria regulate ATP hydrolysis activity. However, taking into account the different ATP binding affinities of the *ɛ* subunit from different organisms, it can be expected that not all bacterial organisms are regulated by ATP binding to the *ɛ* subunit under physiological conditions (in *E. coli*, the *ɛ* subunit has a binding affinity of 22 mM [39]).

ATP binding appears to be a strong influence required to stabilize the hairpin conformation, yet there is still a lack of conclusive evidence on whether ATP synthesis is influenced by the *ɛ* subunit in *E. coli* [69,90,91]. Furthermore, it has been proposed that the CTD of the *ɛ* subunit from *E. coli* [93] and the ATP-bound down state from the *Bacillus* PS3 *ɛ* subunit allow efficient proton coupling to ATP hydrolysis [94]. Together, these data indicate a slightly different working principle in different organisms.

In the authors' view, what is now of intense interest for this micro-field may be the role of the *ɛ* subunit of *P. denitrificans* and other α-proteobacteria—in which it has been shown that the *ɛ* subunit does not inhibit ATP hydrolytic activity, but a novel regulatory subunit, the *ζ* subunit, that harbours an ATP binding site, is present [29]. It will be of interest to examine whether the *ζ* subunit inhibition is influenced by the presence of Mg-ATP.

Considering that biochemical experiments demonstrate that the CTD of the *ɛ* subunit from *C. thermarum* TA2.A1 has a role in the regulation of ATP hydrolysis activity, but not the TA2F_1_ Δ*ɛ*^6A^ mutant [85], it is curious that the WT *ɛ* subunit is found predominantly in the hairpin conformation in the absence of ATP, and that the TA2F_1_ D89A/R92A mutant is in the hairpin conformation despite presumably lacking the ability to bind ATP. It is indeed feasible that the crystal contacts between two neighbouring ATP synthases in the crystal may influence both the TA2F_1_WT and the D89A/R92A mutant structures obtained [45], and is expected to regulate ATP hydrolysis similarly to other bacteria, such as *E. coli* or *Bacillus* PS3. We cannot discount that mechanisms of regulation may be subtle in their diversity. In addition, TA2F_1_ has seemingly little native ATP hydrolysis activity [84], whereas both the *E. coli* and *Bacillus* PS3 enzymes have native ATP hydrolysis activity [95,96], making the *C. thermarum* TA2.A1 a very interesting enzyme to study to aid in unravelling *ɛ* subunit regulatory function. At this point, both the structure and function studies suggest a strong role of ADP inhibition [84] and the *ɛ* subunit as a releasable ‘emergency break’, similar to the suggested role of mammalian IF1 or the alpha-proteobacterial *ζ* subunit. Yet functional studies clearly demonstrate a role in ATP hydrolysis suppression [85], suggesting a dynamic role that may be precluded by crystallization conditions. The lack of resting conformation shown in the NMR studies of the *Bacillus* PS3 *ɛ* subunit support this notion [39]. Taking structural and functional evidence together, a recent claim [45] to have identified the mechanism of the ATP hydrolysis regulation of the *C. thermarum* TA2.A1 F_1_ ATPase would seem premature. While the data presented by the authors were crystal structures of excellent quality, the authors did not reveal the role of the *ɛ* subunit, for which there clearly is a role [85], nor the dynamics of ADP regulation, which is a common mechanism of ATP hydrolysis regulation. Generally, we do not consider observations of TA2F_1_ structure/function to be dismissive of other studies denoting *ɛ* subunit regulatory function in other organisms, but they do add valuable insight into the diversity and tuning involved in ATP synthase regulation.

### 3.2. The 6th antibiotic target space: pathogen ATP synthases as potential drug-targets

Recently, the F_1_F_o_ ATP synthases of certain mycobacterial species have been demonstrated to be promising new drug targets. The drug bedaquiline (BDQ; previously known as compound TMC207) has been shown to be a novel antibiotic compound against tuberculosis [1], and to bind to a purified preparation of *M. smegmatis* and *M. tuberculosis c*-rings, but relatively poorly to the *c* subunit ring mutant *atpE*^A63P^ [2]. To support this, a recent structural study revealed a model of BDQ bound to the *c*-ring of *M. phelei* [97]. Comparisons with the possible binding mode of the drug to other *c*-rings [97] suggest possible reasons for selective binding [98] to various mycobacterial species. Previously, computational models had proposed the drug interacting at the *c*-ring/*a* subunit interface [99], while biochemical and structural data from the Grüber group indicate interactions with *both* the *c*-ring and the *ɛ* subunit [43,100]. Oddly, it would seem that the BDQ binding site and the subunits involved are not clear, as available data are inconsistent. This underlines an inherent risk in the use of crystallography, and the requirement for supporting functional studies. It is duly acknowledged that inhibitors carrying charges may also participate in favourable charge–charge interactions with proteins under favourable pH conditions, which may confound results. Full F_1_F_o_ time-resolved crystallographic affinity constants in direct comparison with affinity constants from pre-steady-state kinetic measurements could be useful tools to clarify the mechanism and binding mode of BDQ. Despite these confounding results, knowledge about the interactions of BDQ with the *c*-ring [97] have allowed an intelligent design approach to construct novel potential antibiotic compounds with a reduced backbone while still binding to the ATP synthase of mycobacteria [101].

Mycobacterial species also appear to have developed several mechanisms to prevent wasteful ATP hydrolysis, an activity that could be fatal for non-growing, infective-phase *M. tuberculosis.* The first regulatory mechanism is Mg^2+^-ADP, and the second may be using the *ɛ* subunit [50]; however, a unique loop in the *γ* subunit may also slow down ATP hydrolysis due to interactions with the *c*-ring [51], and an extended *ɛ* subunit CTD may prevent ATP hydrolysis activity due to interactions with the *γ* subunit [52].

The possible regulatory role of the *γ* subunit in *C. thermarum* TA2.A1 [85] must take into account the different elemental mechanistic steps of the bacterial F_1_ compared with mammalian F_1_, as there may be some possibilities to selectively inhibit the function of the bacterial enzyme, as mitochondrial (bovine) and bacterial (*Bacillus* PS3) ATP synthases have different affinities to various compounds [102]. With this in mind, it would seem a feasible suggestion that novel compounds could be designed which may uncouple respiratory-driven ATP synthesis [103] by interacting with the *c*-ring and the *a* or *γ* subunits. In line with this review, a more bacterial-specific possibility would be to develop drug compounds to modulate the state of the *ɛ* subunit by forcing an unfavourable conformation for the state of bacterial growth (e.g. forcing *M. tuberculosis* to hydrolyse its ATP while in a slow-growth infective state). Further developments of antimicrobial drugs targeting mycobacterial species may also involve compounds that take advantage of interactions of the unique loop in the *γ* subunit with the *c*-ring [51], preventing a rotation in synthesis direction or small organic molecules targeting the interface between subunit *γ* and the extension of the C-terminal domain of subunit *α* [52].

Lastly, while developments have certainly been made towards targeting *M. tuberculosis*, there are several other promising drug targets, such as the F_1_F_o_ ATP synthases of *Trypanosoma brucei* (sleeping sickness) and *Fusobacterium nucleatum*, that all have novel features. The *T. brucei* F_1_F_o_ ATPase has several unique subunits (e.g. P18) of unknown function [104,105]. *Fusobacterium nucleatum* has an F_1_F_o_ ATP synthase that uses sodium as a coupling ion, a common feature in human pathogen ATP synthases. Structural insights into the *c*-ring of this pathogenic organism have recently been obtained and may denote a possible drug target [106]. In all cases, the role of the *ɛ* subunit is undefined, but given its clear regulatory role in catalysis, there is a strong case to support exploring the *ɛ* subunit function more widely.

## Supplementary Material

SI Fig 1

## Data accessibility

This article has no additional data.
